# GraphTransNet: predicting epilepsy-related genes using a graph-augmented protein language model

**DOI:** 10.3389/fphar.2025.1584625

**Published:** 2025-04-01

**Authors:** Junfeng Xie, Wei Li, Hairu You, Dafang Zhang

**Affiliations:** ^1^ College of Computer Science and Electronic Engineering, Hunan University, Changsha, China; ^2^ School of Engineering, The University of Sydney, Sydney, NSW, Australia

**Keywords:** epilepsy-associated interactions, epilepsy diseases, transformer, protein language model, deep learning

## Abstract

**Introduction:** Epilepsy, a complex neurological disorder characterised by recurrent seizures and significant genetic heterogeneity, presents considerable challenges form accurate diagnosis and drug target identification. While traditional genomewide association studies (GWAS) and sequencing technologies have advanced our understanding of epilepsy-related gene targets, they often struggle to identify novel and rare variants crucial for precise diagnosis and targeted drug development. The increasing availability of large-scale genomic data, coupled with the power of deep learning, offers a promising avenue for progress.

**Method:** In this work, we introduce GraphTransNet, a novel hybrid neural network model designed for predicting epilepsy-associated gene targets, with direct implications for improved disease diagnosis and therapeutic target identification. GraphTransNet leverages protein language models (specifically ESM) to generate numerical embeddings from gene sequences. These embeddings are then processed by a novel architecture integrating transformer and convolutional neural network (CNN)components to predict epilepsy-related gene targets.

**Results:** Our results demonstrate that GraphTransNet achieves high accuracy in identifying epilepsy targets, outperforming existing predictive tools in terms of both recall and precision metrics for reliable disease diagnosis and effective drug target identification. Rigorous comparisons with established machine learning methods and other deep learning architectures further underscore the efficacy of GraphTransNet.

**Discussion:** This approach represents a valuable computational tool for advancing epilepsy genetics research, with the potential to contribute to more accurate diagnostic strategies and the discovery of novel drug targets for improved treatment outcomes.

## 1 Introduction

Disease diagnosis prediction and drug target identification are fundamental challenges in modern medicine. Computational approaches have emerged as powerful tools, offering novel perspectives and solutions in these areas. Epilepsy, a chronic and highly heterogeneous neurological disorder, affects approximately 50 million people worldwide, ranking among the most prevalent neurological conditions. Characterized by recurrent, unprovoked seizures stemming from abnormal electrical brain activity, epilepsy significantly impacts patients’ lives (Fisher et al., 2014; Thurman et al., 2011). Despite medical progress, around 30% of epilepsy patients suffer from drug-resistant epilepsy (DRE), which not only deteriorates their quality of life but also heightens the risk of severe complications like sudden unexpected death in epilepsy (SUDEP) (Holger, 2020; Devinsky et al., 2016). In this context, computational methods hold great potential for understanding the genetic basis of epilepsy, which is crucial for identifying new therapeutic targets, especially for DRE patients.

The genetic landscape of epilepsy is incredibly intricate, involving diverse genetic variations such as single nucleotide polymorphisms (SNPs), copy number variations (CNVs), and rare *de novo* mutations (Ellis et al., 2020). Over 500 genes have been associated with epilepsy, covering ion channels, neurotransmitter receptors, and synaptic proteins, all vital for maintaining neuronal excitability and synaptic transmission Scheffer and Berkovic (1997); Myers and Mefford (2015); Liang et al. (2025). Computational techniques could potentially untangle this complexity. However, the genetic architecture of epilepsy remains incompletely understood, with many cases lacking a genetic explanation. For example, generalized epilepsy often has polygenic origins, while rare monogenic forms like Dravet syndrome and Lennox-Gastaut syndrome are linked to mutations in specific genes such as SCN1A and GABRG2. These genetic patterns pose challenges that computational methods are uniquely positioned to address, as they can analyze large datasets and identify hidden relationships among genes (Ji et al., 2020b; 2025).

The advent of next-generation sequencing (NGS) technologies, including whole-exome sequencing (WES) and whole-genome sequencing (WGS), has transformed epilepsy research (Daniel et al., 2024; DC et al., 2013; Consortium et al., 2013). These methods have allowed the identification of rare mutations and genetic risk factors previously undetected by traditional techniques. Transcriptomic studies and epigenomic profiling have also shed light on how regulatory elements, non-coding RNAs, and chromatin modifications contribute to epilepsy pathogenesis. However, the vast amount of data generated by NGS presents significant analytical hurdles. Computational approaches are essential for managing and interpreting this data. In particular, distinguishing pathogenic variants from benign ones is a complex task. Conventional alignment-based methods often face limitations in identifying novel or divergent genetic variants due to their reliance on reference databases (Wang et al., 2023; Wei et al., 2024), highlighting the need for advanced computational algorithms to uncover new epilepsy-associated genes.

Emerging computational approaches, especially machine learning and deep learning, offer promising solutions to these challenges (Kenji, 2016). Deep learning models, particularly genomic language models trained on large scale sequencing data, are designed to extract complex patterns and relationships from genetic information (Ji et al., 2020a; 2024). These models utilize the contextual information within nucleotide sequences to capture subtle genetic signals related to diseases. For example, they can analyze the sequence data to predict the likelihood of a gene being associated with epilepsy. However, their accuracy is currently constrained by limited training data, a significant obstacle, especially for rare and heterogeneous disorders like epilepsy. This limitation underscores the need for innovative computational strategies to enhance the performance of these models in disease diagnosis prediction and drug target identification.

**FIGURE 1:**
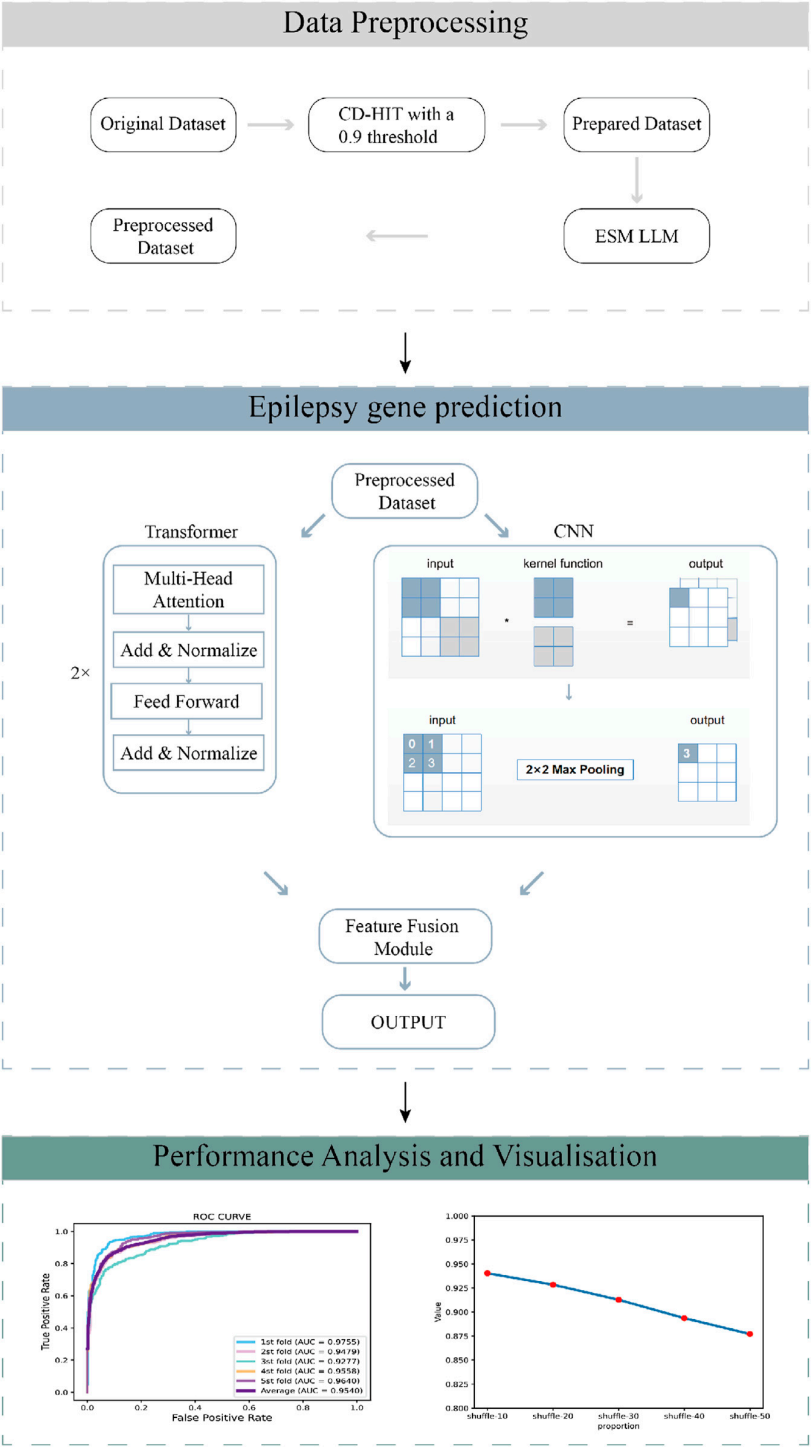
The workflow of the GraphTransNet method, which has three main steps: (1) Data preprocessing filters sequences with over 90% similarity using a 0.9 CD-HIT threshold, then extracts numerical features using the ESM protein language model. (2) Epilepsy gene prediction uses Transformer and CNN networks for feature extraction, combines the features, and performs binary classification. (3) Performance analysis and visualization evaluate and display the predictive performance of GraphTransNet.

To overcome these limitations and improve disease diagnosis prediction and drug target identification in the context of epilepsy, we propose EffuTCN, a novel framework. EffuTCN combines pre-trained protein language models with Transformer (Ashish et al., 2017) and CNN-based neural networks (Lecun et al., 1998). By leveraging this combination, EffuTCN aims to enhance the prediction of epilepsy-related targets, which is crucial for both diagnosing the disease at a genetic level and identifying potential drug targets. The flowchart of EffuTCN is presented in Figure 1. EffuTCN uses the gene sequence data as inputs to the protein language model ESM (Lin et al., 2023) to obtain corresponding numerical features. These features are then fed into a neural network model for epilepsy gene prediction. This innovative approach not only addresses the drawbacks of traditional methods, but also provides a new avenue for a more in-depth understanding of the genetic structure of epilepsy, ultimately contributing to more effective prediction of disease diagnosis and drug target identification.

## 2 Materials and methods

### 2.1 Evaluation Method

We used multiple metrics to evaluate the prediction performance of GraphTransNet for epilepsy genes, specifically, in addition to accuracy (ACC), precision (Prec), recall (Rec), f1 score (F1), Matthews correlation coefficient (MCC), AUPR (area under the precision-recall curve) and AUC (area under the ROC curve, which is plotted by the false-positive rate (FPR) and true-positive rate (TPR)). They are calculated using the following Equations 1-6:

$$accuracy=\frac{TP+TN}{TP+FP+TN+FN}$$ (1)

$$precision=\frac{TP}{TP+FP}$$ (2)

$$TPR=recall=\frac{TP}{TP+FN}$$ (3)

$$F1\text{\_}score=\frac{2\times precision\times recall}{precision+recall}$$ (4)

$$MCC=\frac{TP\times TN-FP\times FN}{\sqrt{\left(TP+FP\right)\left(TP+FN\right)\left(TN+FP\right)\left(TN+FN\right)}}$$ (5)

$$FPR=\frac{FP}{FP+TN}$$ (6)

Where TP denotes the number of actual labels that are positive and predicted to be positive as well, FP denotes the number of actual labels that are negative and predicted to be positive, and the same for TN and FN.

### 2.2 Data preprocessing

Several databases provide information on epilepsy-related genes, forming the foundation for building a prediction model. We collected epilepsy-related genes from recent databases and research papers and retrieved their sequences from the UniProt database, resulting in a total of 6,452 sequences. Detailed information about these databases is presented in Table 1.

**TABLE 1:** The detailed information for the positive benchmark dataset about epilepsy-associated genes.

Database	Amount
GWAS (https://www.ebi.ac.uk/gwas/home) (Elliot et al., 2023)	86
ClinVar (https://www.ncbi.nlm.nih.gov/clinvar/) (Landrum et al., 2016)	184
Uniprot (https://www.uniprot.org/) (Consortium, 2015)	487
EpilepsyGene (http://www.wzgenomics.cn/EpilepsyGene/) (Xia et al., 2015)	499
Genes4Epilepsy (github.com/bahlolab/genes4epilepsy) (Oliver et al., 2023)	955
Wang et al. (Jie et al., 2017)	977
DisGeNET (https://www.disgenet.org/) (Janet et al., 2016)	3264
Total	6452

It is important to note that the extracted sequences included duplicates and sequences with high similarity. To address this, we applied CD-HIT with a 0.9 threshold to remove sequences with more than 90% identity to others. Additionally, we reviewed and corrected any erroneous amino acid representations in the sequences.

For negative samples, we randomly selected an equal number of protein sequences from the UniProt (Consortium, 2015) database, ensuring that none overlapped with the positive sample dataset. This step balanced the dataset with an equal number of positive and negative samples. After obtaining equal numbers of positive and negative sample sequences, we processed these sample sequences through ESM (Lin et al., 2023) to obtain the digital features of each sample.

To assess the robustness of the prediction model, we conducted five-fold cross-validation. Specifically, in each fold, 20% of the initial dataset was randomly selected as the validation set, while the remaining 80% was used as the training set to develop the model. This process was repeated five times to ensure comprehensive evaluation.

### 2.3 Protein-protein interaction prediction based on transformer and CNNs

Transformer is a deep learning model architecture introduced by Vaswani et al. (2017), widely applied in natural language processing (NLP) and other fields (Ashish et al., 2017). Its core innovation is the self-attention mechanism, which captures long-range dependencies in sequence data, addressing the vanishing gradient problem encountered by traditional recurrent neural networks (RNNs) in long sequences (Rumelhart et al., 1986). The Transformer employs multi-head attention to perform parallel computation, significantly improving training efficiency. It does not rely on sequential processing, instead preserving order information through positional encoding, offering strong parallelization capabilities and scalability. This architecture has become the foundation of many modern NLP models, and has also achieved success in image processing and other domains.

Convolutional Neural Networks (CNNs) are a class of deep learning models designed to process data with a grid-like structure, such as images (Lecun et al., 1998). CNNs are composed of multiple layers, including convolutional layers, pooling layers, and fully connected layers. The convolutional layers apply filters (or kernels) to the input data, allowing the network to automatically learn spatial hierarchies of features, such as edges, textures, and shapes. Pooling layers downsample the data, reducing its dimensionality and computational complexity while retaining important features. CNNs have been particularly successful in computer vision tasks, such as image classification, object detection, and segmentation, due to their ability to learn complex patterns and generalize well to unseen data.

In GraphTransNet, we used both Transformer and CNN to predict whether a gene was related to epilepsy. Specifically, we used their numerical features as input to the Transformer encoder and the CNN, respectively. Finally, the features obtained from both were fused through the self-attention fusion module, followed by a binary classification task.

Assuming the input is X with shape $\left(num_{s}ample,num_{f}eature\right)$ , when we use the Transformer for feature extraction, we first transform X into $X^{\prime }$ with shape $\left(1,num_{s}ample,num_{f}eature\right)$ . Then, the multi-head self-attention module processes $X^{\prime }$ , and its computation can be described by the following Equations 7-20:

$$Q_{i}=X^{\prime }W_{i}^{Q}$$ (7)

$$K_{i}=X^{\prime }W_{i}^{K}$$ (8)

$$V_{i}=X^{\prime }W_{i}^{V}$$ (9)

Where Q is the query matrix, K is the key matrix, V is the value matrix, $W_{i}^{Q}$ , $W_{i}^{K}$ , $W_{i}^{V}$ are trainable weight matrices, i represents the *i*th self-attention head.

Attention weights are then calculated using scaled dot product attention. For each head i, the dot product of the query and key is first calculated, then scaled and the softmax function is applied as follows:

$$A_{i}=softmax\left(\frac{Q_{i}K_{i}^{T}}{\sqrt{d_{k}}}\right)$$ (10)

the formula for $d_{k}$ is as follows:

$$d_{k}=\frac{d_{\mathrm{model}}}{nhead}$$ (11)

where d_model is the feature dimension of the model, nhead is the number of attention heads.

Then use the attention weight matrix $A_{i}$ to add the weights matrix $V_{i}$ , the formula is as follows:

$$O_{i}=A_{i}V_{i}$$ (12)

$O_{i}$ is the output of each header.

Subsequently, we concat the output of all the headers according to the last dimension, the formula is as follows:

$$O=concat\left(O_{1},O_{2},\ldots \ldots ,O_{\mathrm{nhead}}\right)$$ (13)

Finally, a linear transformation matrix $W^{O}$ is used to transform the spliced output to obtain the final multi-head self-attention output:

$$O_{\mathrm{final}}=O_{\mathrm{concat}}W^{O}$$ (14)

For the calculation of the CNN part, we convert the input X to the shape of ( batch_size , sq_num_feature , sq_num_feature ) for processing, where sq_num_feature is the square root of num_feature . For this data, we only need to set the number of channels to 1. Thus, each convolution operation can be expressed as follows:

$$Y_{o,i,j}=\sigma \left(\overset{C_{in}}{\underset{c=1}{\sum }}\overset{K_{h}-1}{\underset{p=0}{\sum }}\overset{K_{w}-1}{\underset{q=0}{\sum }}W_{o,c,p,q}\cdot X_{c,i+p,j+q}+b_{o}\right)$$ (15)

where $X_{c,i,j}$ represents the pixel value at position (i,j) of the cth channel in the input feature map, $C_{in}$ represents the number of channels, o represents the oth output channel, $K_{h}$ and $K_{w}$ represent the height and width of the convolution kernel, p and q are the offset indexes of the convolution kernel, $b_{o}$ is the bias term of the oth output channel, and $W_{o,c,p,q}$ is the weight of the convolution kernel. σ is the activation function, here it means ReLU, its formula is as follows:

$$f\left(x\right)=max\left(0,x\right)$$ (16)

The convolution operation is followed by the addition of a maximum pooling layer with the following equation:

$$Y_{i,j,k}=\overset{P}{\underset{p=0}{\max}}\overset{Q}{\underset{q=0}{\max}}X_{k,i+p,j+q}$$ (17)

where P and Q are the height and width of the pooling window.

Subsequently, for the feature fusion module, we assume that the features obtained after Transformer and CNN processing are T and C , respectively. we first compute the attention weights with the following formula:

$$a=\text{softmax}\left(W_{2}\cdot \tanh\left(W_{1}\cdot \left.\left[T;C\right]\right]+b_{1}\right)+b_{2}\right)$$ (18)

Among them, W and b are learnable parameters, and [T;C] means concatenating T and C . Both softmax and tanh are activation functions.

After the above processing, the attention weight $a=\left[a_{c},a_{t}\right]$ is obtained, and then the feature is weighted by the attention weight, and the formula is:

$$T^{\prime }=a_{t}\cdot W_{t}\cdot T,C^{\prime }=a_{c}\cdot W_{c}\cdot C$$ (19)

Next, concatenate them to get the fused features, namely, the following formula:

$$F_{\mathrm{fused}}=\left[T^{\prime };C^{\prime }\right]$$ (20)

Finally, the binary classification task is performed through the fully connected layer.

## 3 Results and discussion

### 3.1 Evaluation of prediction performance

**FIGURE 2:**
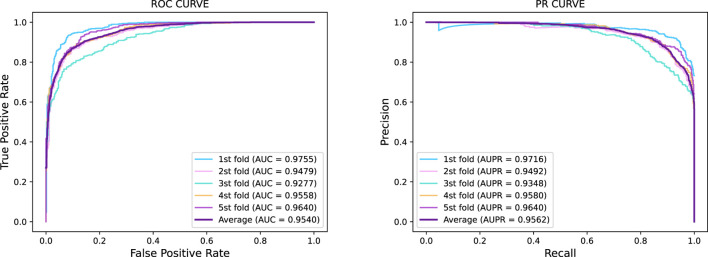
Five-fold cross-validation ROC curve and PR curve of GraphTransNet predicting epilepsy genes.

In this section, we used five-fold cross validation to divide the data into five parts, where 4/5 of the data was used for training and 1/5 was used for validation. We used the metrics mentioned in the Evaluation Method to evaluate the performance of GraphTransNet. The results are shown in Table 2. In addition, in order to more visually demonstrate the performance of GraphTransNet on the task of predicting epilepsy genes, we plotted the ROC and PR curves for five-fold cross-validation, as shown in Figure 2. As we observed, the AUC and AUPR were higher than 0.92 in all five validations, which also demonstrated the stability of GraphTransNet on the task of predicting epilepsy genes.

**TABLE 2:** The average performance of GraphTransNet obtained through five-fold cross-validation.

Dataset	Acc. (%)	Prec. (%)	Rec. (%)	F1. (%)	MCC(%)	AUPR(%)	AUC(%)
dataset	88.16	89.98	85.91	87.84	76.48	95.55	95.42

### 3.2 Ablation study

#### 3.2.1 Ablation experiment of feature fusion module

In this section, we will compare the performance of GraphTransNet using the feature fusion module with that without the module. The model without the feature fusion module directly concatenates the features extracted by Transformer and CNN and then performs the binary classification task. The results are shown in Table 3. We can observe that the GraphTransNet with the feature fusion module has at least one percent improvement in all metrics.

**TABLE 3:** Epilepsy gene prediction results under five-fold cross validation using GraphTransNet with and without the feature fusion module.

Dataset	Acc. (%)	Prec. (%)	Rec. (%)	F1. (%)	MCC(%)	AUPR(%)	AUC(%)
with fusion	88.16	89.98	85.91	87.84	76.48	95.55	95.42
without fusion	86.40	88.45	84.01	85.98	73.17	94.21	93.99

#### 3.2.2 Ablation experiment of transformer module

In this part, we will investigate the improvement of the GraphTransNet performance by the Transformer module, specifically, we will set up another set of models with only CNN module for the task of binary classification of epilepsy genes. In order to ensure the only variable, we compare it with GraphTransNet without feature fusion module. The results are shown in Table 4. We can observe that almost all of them achieve at least a two percent improvement in performance, except for the recall metric, which also demonstrate the importance of the Transformer module in the GraphTransNet model.

**TABLE 4:** Epilepsy gene prediction results under five-fold cross validation using GraphTransNet with and without the Transformer module.

Dataset	Acc. (%)	Prec. (%)	Rec. (%)	F1. (%)	MCC(%)	AUPR(%)	AUC(%)
with Transformer	86.40	88.45	84.01	85.98	73.17	94.21	93.99
without Transformer	83.71	83.31	84.46	83.83	67.51	91.30	91.59

#### 3.2.3 Ablation experiment of CNN module

In this section, we will investigate the improvement in GraphTransNet performance brought by the CNN module. Specifically, we will set up another model with only the Transformer module for the binary classification task of epilepsy-related genes. As in the previous experiment, we compare with the model that directly concatenates the features extracted by Transformer and CNN. The results are shown in Table 5. It can be observed that there is at least one percent performance improvement in almost all metrics.

**TABLE 5:** Epilepsy gene prediction results under five-fold cross validation using GraphTransNet with and without the CNN module.

Dataset	Acc. (%)	Prec. (%)	Rec. (%)	F1. (%)	MCC(%)	AUPR(%)	AUC(%)
with CNN	86.40	88.45	84.01	85.98	73.17	94.21	93.99
without CNN	85.65	88.19	82.48	85.11	71.61	93.66	93.40

#### 3.2.4 Ablation experiment on the number of encoder layers in the transformer module

In this section, we further investigate whether the number of encoder layers in the transformer module affects the prediction performance of epilepsy genes. Specifically, we vary the number of encoder layers in the transformer module in GraphTransNet in the expectation of finding an optimal number of encoder layers. The results are shown in Table 6. We can observe that when the number of encoder layers is 3, the performance is optimal across almost all metrics. However, as the number of encoder layers increases further, the performance gradually declines.

**TABLE 6:** Epilepsy gene prediction results of GraphTransNet with different encoder layers under five-fold cross-validation. “encoder-n” means that there are n encoder layers.

Dataset	Acc. (%)	Prec. (%)	Rec. (%)	F1. (%)	MCC(%)	AUPR(%)	AUC(%)
encoder-1	83.18	87.59	77.41	81.96	67.06	92.81	92.38
encoder-2	88.16	89.98	85.91	87.84	76.48	95.55	95.42
encoder-3	89.88	91.02	88.51	89.66	79.93	96.43	96.34
encoder-4	89.32	88.86	89.96	89.37	78.71	95.64	95.69
encoder-5	85.21	90.92	78.06	83.78	71.33	94.00	93.86
encoder-6	81.99	83.54	81.14	81.95	64.60	92.15	91.63

### 3.3 Investigation of prediction with incomplete sequences

**FIGURE 3:**
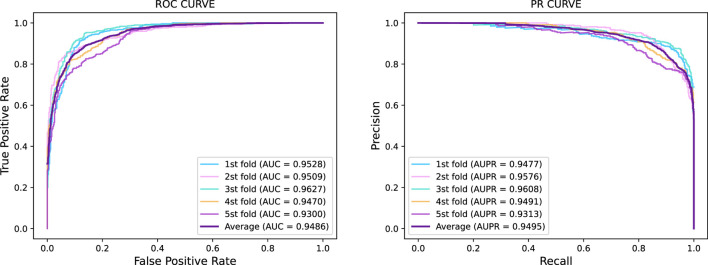
Five-fold cross-validation ROC curve and PR curve of GraphTransNet predicting epilepsy genes with incomplete sequences.

In this section, we investigated the prediction performance of GraphTransNet for epilepsy genes with incomplete sequences. Specifically, we intercepted the first 500 amino acids of the sequence corresponding to each gene to explore the prediction performance of GraphTransNet for incomplete sequences. The results are shown in Table 7. In addition, we plotted the ROC and PR curves under five-fold cross validation, as shown in Figure 3. We can observe that the performance degradation on almost all metrics is less than one percent (compare with Table 2). Furthermore, as shown in Figure 3, both AUC and AUPR exceed 0.93 across five-fold cross-validation, demonstrating the stability of GraphTransNet in predicting epilepsy genes with incomplete sequences.

**TABLE 7:** Epilepsy gene prediction results of GraphTransNet for incomplete sequences under 5-fold cross validation.

Dataset	Acc. (%)	Prec. (%)	Rec. (%)	F1. (%)	MCC(%)	AUPR(%)	AUC(%)
dataset	87.11	88.19	85.83	86.89	74.41	94.93	94.87

### 3.4 Investigation on the prediction performance in the presence of noise

**FIGURE 4:**
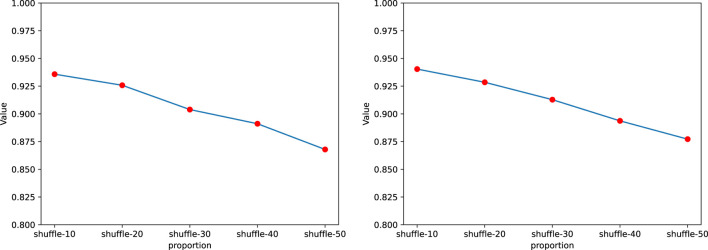
The average AUC and AUPR of GraphTransNet in predicting epilepsy genes under different shuffle ratios.

**FIGURE 5:**
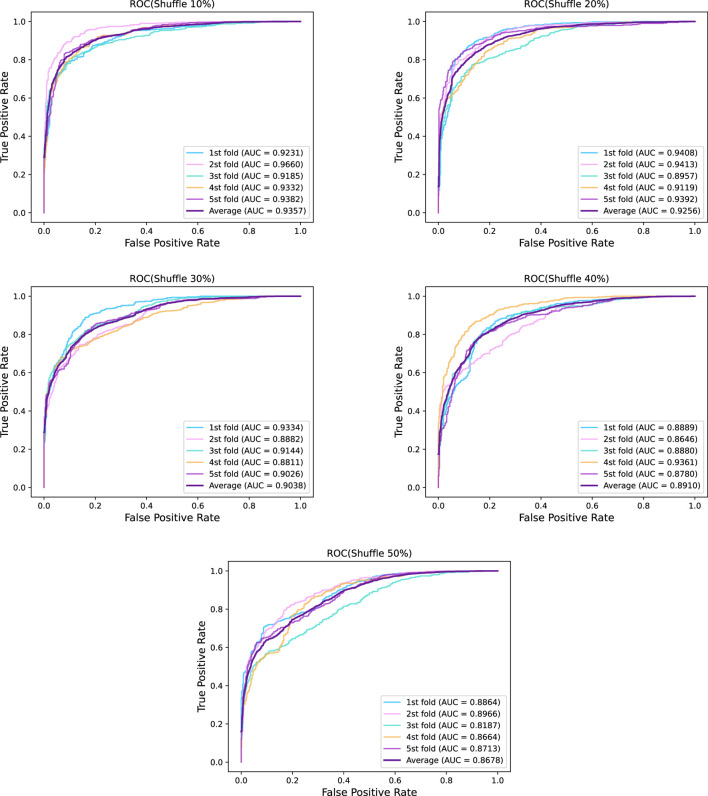
ROC of five-fold cross validation of GraphTransNet at different shuffle ratios.

**FIGURE 6:**
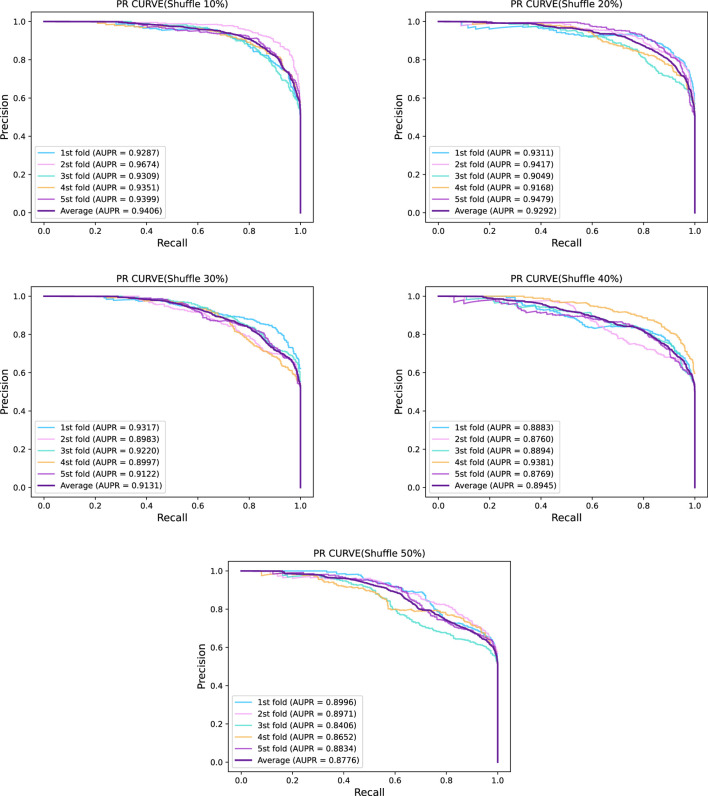
PR Curves of five-fold cross validation of GraphTransNet at different shuffle ratios.

In this section, we introduced noise into the sequences to explore its impact on the performance of GraphTransNet. Specifically, we randomly selected certain continuous segments from the complete sequences and shuffled the selected segments to simulate noise. We conducted a total of five experiments, where we randomly selected and shuffled segments accounting for 10%–50% of the length of the complete sequences. Each experiment was performed using five-fold cross-validation, and the average AUC and AUPR obtained from the five-fold cross-validation were visualized, as shown in Figure 4. We can observe that as the shuffle ratio increases, both AUC and AUPR decrease. However, when the shuffle ratio reaches 50%, both AUC and AUPR remain above 0.85. Additionally, to demonstrate the stability of GraphTransNet in terms of performance at different shuffle ratios, we plotted the ROC and PRC curves from five-fold cross-validation for each shuffle ratio. As shown in Figures 5, 6. As observed, when the shuffle ratio is below 50%, the AUC and AUPR for each validation are consistently above 0.85. Even with a shuffle ratio of 50%, both AUC and AUPR remain above 0.8 in every validation.

## 4 Conclusion

In this study, we introduced GraphTransNet, a deep learning framework specifically designed to predict epilepsy-related Protein-Protein Interactions (PPIs)—a critical advancement for uncovering disease mechanisms and accelerating therapeutic discovery. By integrating structural insights from protein language models with a hybrid Transformer-CNN architecture, our model addresses the unique challenges of mapping dynamic PPIs underlying epileptogenesis. GraphTransNet’s ability to simultaneously resolve local interaction interfaces (e.g., ion channel binding motifs) and global PPI network dependencies (e.g., synaptic complex formations) represents a paradigm shift in computational epilepsy research.

Through rigorous five-fold cross-validation and comprehensive benchmarking, we demonstrated GraphTransNet’s superior performance in PPI prediction accuracy, particularly for noisy biological datasets where traditional methods falter. The model’s robustness stems from its dual capability: the Transformer component identifies long-range structural determinants of PPIs, while CNNs decode evolutionarily conserved interaction patterns within protein sequences. Our ablation studies confirmed that both architectural elements synergistically enhance PPI prediction fidelity, disproving trivial feature reliance and validating the design’s biological relevance.

Beyond technical innovation, GraphTransNet offers transformative practical value. Its computational efficiency enables large-scale PPI network mapping across diverse hardware environments, democratizing access for both research and clinical applications. This capability is particularly crucial for epilepsy, where pathogenic PPIs often involve rare variants in multi-protein complexes like GABA receptors or potassium channel clusters. By systematically prioritizing therapeutically actionable PPIs, our framework bridges the gap between genomic findings and mechanistic insights—a persistent bottleneck in precision neurology.

The clinical implications are profound: GraphTransNet’s PPI-centric predictions provide a roadmap for repurposing existing drugs (e.g., those targeting NMDA receptor interactions) and developing novel biologics to disrupt seizure-driving PPIs. Furthermore, its ability to interpret variant-induced PPI perturbations enhances diagnostic precision for drug-resistant epilepsy cases. As the first model explicitly optimized for epilepsy-specific PPIs, GraphTransNet establishes a new standard for computational target discovery, with potential extensions to other neurological disorders governed by dysregulated interaction networks.

## Data Availability

The original contributions presented in the study are included in the article/supplementary material, further inquiries can be directed to the corresponding author.
